# A Public Medical Service in Being

**Published:** 1918-06-15

**Authors:** 


### A PUBLIC MEDICAL SERVICE IN BEING.

### Leicester's Useful Example.

Whilst others have been discussing the extension of the provision of medical treatment to the poor by the State, the medical men of Leicester have organised a Public Medical Service, which shows how much can be done with advantage to the doctors and the community without waiting for any special legislation. The Service started in January 1913, when, owing to the closing of the Leicester Provident Dispensary, its members, chiefly women and children unable to pay ordinary medical fees, lost the medical attendance provided for them up to then. All the doctors in Leicester joined the Service, which is under the control of a board of management elected annually. There are a central dispensary, with consulting-rooms, and eight branch dispensaries in various parts of the town, with consulting-rooms at five of them. Prescriptions are made up by qualified dispensers at the dispensaries or by the chemists in the towns. The association has published a very useful and practical Pharmacopœia containing over 150 formulæ. The doctors can be consulted either at the dispensaries or at their homes. The Service provides dental treatment and specialist treatment for eye, nose, ear, and throat cases on the payment of very moderate fees, not only for members, but for children sent by the Education Committee. It has also arranged for the medical attendance on patients whose doctors are absent on military service. Members requiring hospital treatment can get it at the Faire Hospital, containing twenty-seven beds, on payment of reduced charges for nursing and maintenance, and of an arranged fee for operation and the attendance, if they wish it, of their own doctors. For all these advantages adult members pay 2d. per week, and their children under fourteen years of age 1d. per week. If there is no adult member the first child is charged 1½d. per week, but no family is charged for more than four children. These payments give the right to ordinary medical attendance, medicine, dressings, etc., at the dispensaries, or at the member's home if he is too ill to go out. These privileges become due one month after application, but if the applicant is actually ill he can obtain them at once by the payment of an additional sick fee of 10s. Members have a choice from a panel of sixty doctors, and can change their doctors quarterly or more frequently if they move from one part of the town to another. All the friendly societies in Leicester make arrangements for the medical treatment of their uninsured members through the Public Medical Service, and the local Board of Guardians, with the approval of the Local Government Board, sends its outdoor sick-poor to it for the dispensing of medicines. The Service gives gratuitous medical treatment to members of the Institution for the Welfare of the Blind, of the Cripples' Guild, and of the Home for Feeble-minded Girls, and, on special terms, to the dependants of sailors and soldiers. The fact that no complaint has ever been received speaks for the efficiency of the Service, and its success is shown by the following figures:—The number of members exceeds 42,000; school clinic, new cases in 1917, 2,242; prescriptions dispensed annually, about 190,000.

The board of management is considering extensions and improvements of the Service, especially the provision of consultants and of departments for *x*-ray and electro-therapeutics, for pathology, for skin diseases, for diseases of women and children, and for nervous diseases. This short review shows that the medical men of Leicester have solved a difficult problem to the satisfaction of themselves and of the uninsured poor in the town. Obviously the weakness of the scheme lies in the insufficiency of its arrangements for special and institutional treatment and in its failure to provide trained nursing for the sick. One cannot expect the poor for whom this scheme caters to find the money for all these requirements of a perfect scheme. The State or charity must give the funds, and no doubt charity does so to a large extent through the voluntary hospitals. Any scheme for the reorganisation of medical assistance could link up easily with this Service, supplying its deficiencies without undermining the independence of its members. All honour to the medical men of Leicester for their public-spirited and pioneer work. Their success should spur the medical men of other towns to follow in their steps.

